# The Role of Clinical Glyco(proteo)mics in Precision Medicine

**DOI:** 10.1016/j.mcpro.2023.100565

**Published:** 2023-05-09

**Authors:** Yuri van der Burgt, Manfred Wuhrer

**Affiliations:** Center for Proteomics and Metabolomics, Leiden University Medical Center, Leiden, The Netherlands

**Keywords:** glycomics, glycoproteomics, multimarker, biomarker, quantification, clinical, diagnosis, therapy, lab-developed test

## Abstract

Glycoproteomics reveals site-specific O- and N-glycosylation that may influence protein properties including binding, activity, and half-life. The increasingly mature toolbox with glycomic and glycoproteomic strategies is applied for the development of biopharmaceuticals and the discovery and clinical evaluation of glycobiomarkers in various disease fields. Notwithstanding the contributions of glycoscience in identifying new drug targets, the current report is focused on the biomarker modality that is of interest for diagnostic and monitoring purposes. To this end, it is noted that the identification of biomarkers has received more attention than the corresponding quantification. Most analytical methods are very efficient in detecting large numbers of analytes, but developments to accurately quantify these have so far been limited. In this perspective, a parallel is made with earlier proposed tiers for protein quantification using mass spectrometry. Moreover, the foreseen reporting of multimarker readouts is discussed to describe an individual’s health or disease state and their role in clinical decision-making. The potential of longitudinal sampling and monitoring of glycomic features for diagnosis and treatment monitoring is emphasized. Finally, different strategies that address the quantification of a multimarker panel are discussed.

## Highlights

• Glycoproteomics for diagnostic and monitoring purposes.
• Quantification of glycobiomarkers in clinical labs.
• Multimarker readouts and their role in clinical decision-making.

## Graphical Abstract

**Figure:**
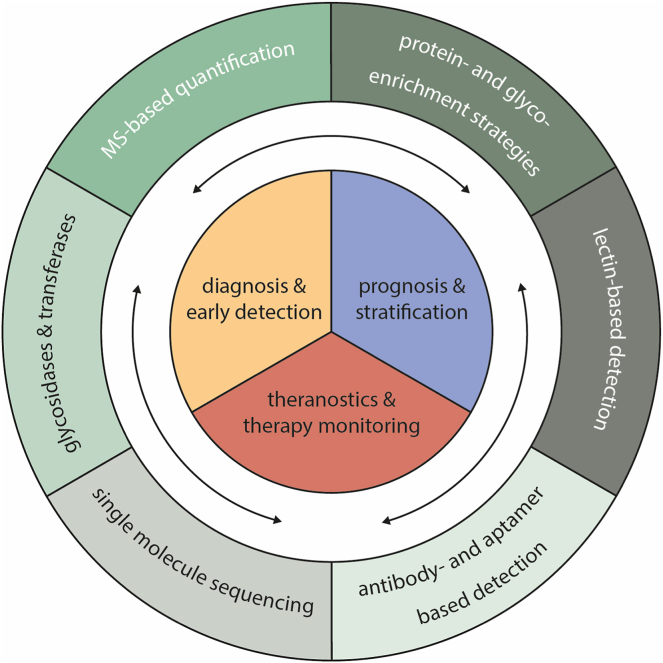

## In Brief

The increasingly mature toolbox with glycomic- and glycoproteomic strategies is applied for the development of biopharmaceuticals and the discovery and clinical evaluation of glycobiomarkers in various disease fields. In this perspective on clinical glycoproteomics, a parallel is made with earlier proposed tiers for protein quantification using mass spectrometry. Multimarker readouts are foreseen with the potential for longitudinal sampling and monitoring of glycomic features for diagnosis and treatment monitoring. The importance of accurate quantification of a multimarker panel is discussed.

## Protein Glycosylation and Roadmaps for Glycoscience

Comprehensive protein analysis requires careful attention to post-translational modifications (PTMs) (1). One of the most widely occurring and arguably the most complex PTM is the covalent attachment of glycan structures to a protein, affecting protein stability, fate, and function (2, 3). Glycan structures are impacted by the cellular environment including pH, enzyme, and substrate availability (4, 5). Other biomolecules with covalently bound oligosaccharides are proteoglycans (protein with one or more glycosaminoglycan (GAG) polysaccharides) and glyco(sphingo)lipids (6, 7). In general, glycans are assembled sequentially by the concerted actions of multiple enzymes, resulting in a heterogeneous array of related structures with different functions. The structures are impacted by the cellular environment including pH and the availability of both enzyme (such as a glycosyltransferase) and substrate (such as an activated monosaccharide). The resulting structures are not directly genetically encoded; however, a defect in glycosyltransferase genes can translate into congenital disorders of glycosylation (CDGs) (8, 9, 10, 11, 12). In general, dysregulation of (protein) glycosylation results in specific aberrations in glycan structures, and these may serve as clinical biomarkers for diagnosis and treatment monitoring (13). Such structures as well as altered glycomic profiles potentially hold promise as disease-specific biomarkers and may aid in the stratification of patients (14). In the early days of omics, the clinical potential of glycans as diagnostic markers and therapeutic targets was noticed and has led to the advancement of glycoscience into clinical applications (15, 16, 17). This clinical potential was emphasized in 2012 in the Roadmap on Transforming Glycoscience (8). This roadmap, published by the national academy of sciences, a committee of US glycoscientists appreciated the role of glycans in health and disease as well as their clinical potential in the detection and treatment of disease. Notably, it was concluded that “the tools for realizing this potential are not available yet” and in addition, concerns were raised with regard to a lack of visibility, awareness, and education. It was foreseen that collaborative efforts were needed to establish an analytical toolkit for glycosciences. A decade later, it is encouraging to see that the high-throughput glyco-analytical tools have been largely established and are ready for clinical evaluation (18, 19, 20, 21, 22). Health and disease likewise received ample attention in the Roadmap for Glycoscience in Europe in 2020, summarizing how glycomic and glycoproteomic analyses are applied in the development of biopharmaceuticals and how glycoscience can contribute to personalized medicine in the fields of viral and bacterial infections, cancer, diabetes and immune diseases (23). Site-specific glycosylation determines protein properties such as binding, activity, and half-life. Dissecting site-specific protein glycosylation may come with therapeutic and diagnostic implications with a large potential for personalized medicine (24).

## Scope of This Perspective

In this perspective, recent progress in clinical glycoproteomics applications is presented, and a path is outlined for further translation into precision medicine with a focus on patient stratification and diagnosis. The foreseen change from a single biomarker readout to a multimarker one for diagnostic and therapy monitoring purposes will be addressed. Select glycoproteomics applications for various diseases will be exemplified without aiming for a comprehensive overview.

## Clinical Glycoproteomics Approaches

**Fig. 1:**
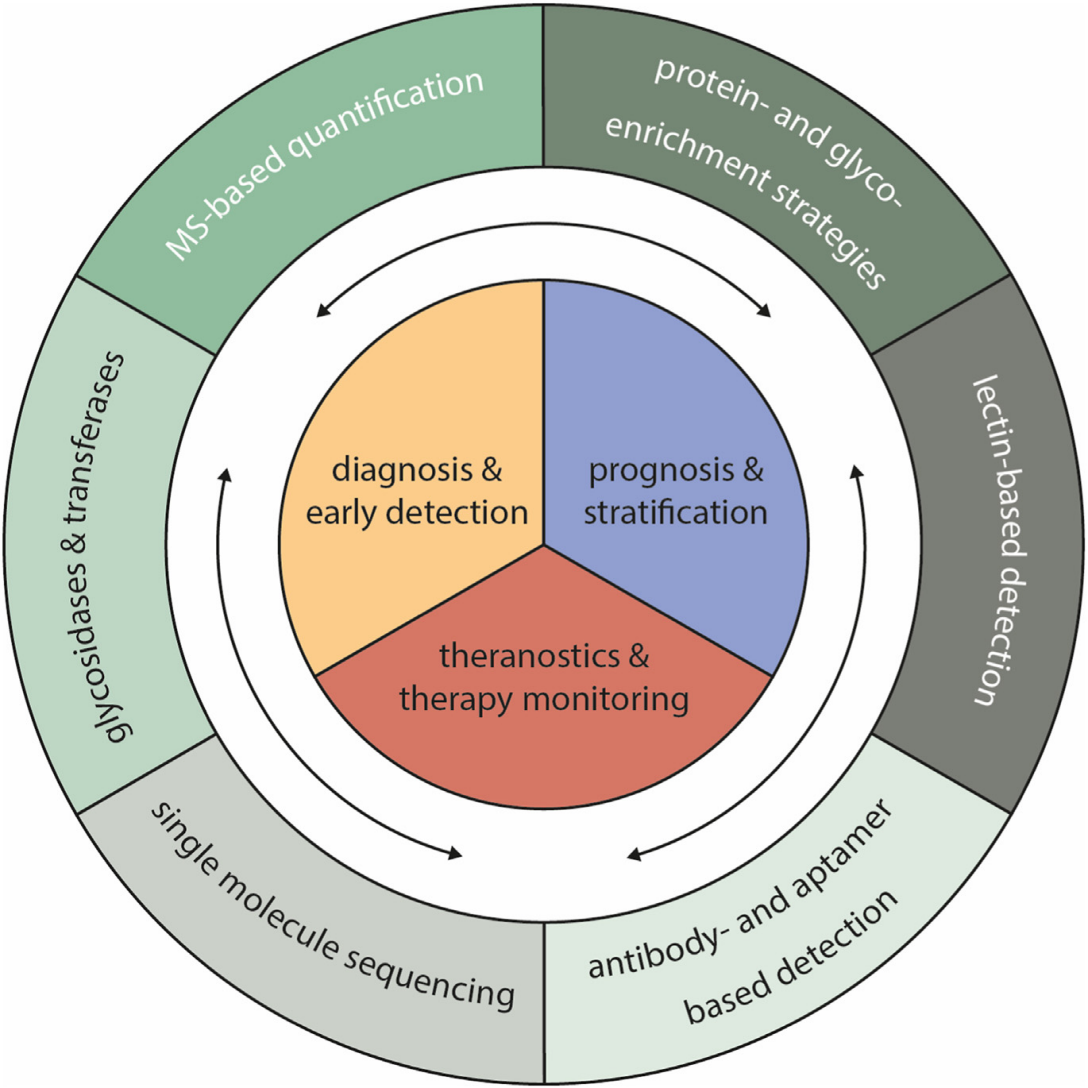
**Analytical glycoproteomics with potential applications in clinical laboratories.** The six different strategies that measure glycan markers in the outer ring can be turned to address one of the clinical parameters in the inner circle.

The glyco-analytical strategies that are used for clinical applications have been overviewed elsewhere and include total serum N-glycome analysis *via* N-glycan release, protein O-glycosylation analysis, proteoglycan (GAG) analysis, and glycopeptide-based strategies with or without prior glycoprotein extraction (18, 19, 20, 21, 25, 26, 27). The first three approaches result in a general overview of glycosylation profiles and—differences in an—omics-based manner with relative quantifications. Such profiles provide a basis for further in-depth characterization and more accurate quantification of biomarker candidates. The latter can be done *via* glycopeptide-based strategies that consist of simultaneous identification of proteins and corresponding glycosylation features with inherent protein- and site-specificity of O- and N-glycopeptide analysis, albeit that identifications turn more tedious. Commonly, data are evaluated by considering each glycan- or glycopeptide signal independently or by grouping glycan structures that have similar structural properties into derived glycosylation traits (18, 19, 28). For the purpose of smoothening identifications, a large variety of glycopeptide enrichment strategies has been applied in glycoproteomics studies (22). Alternatively, glycoproteins such as transferrin and immunoglobulins can be identified and quantified and their glycosylation profile assessed in their intact or semi-intact form by using ultrahigh resolution mass spectrometry (MS). In all glyco-analytical strategies, mass spectrometry (MS) combined with different types of chromatography has dominated innovations. However, the translation into clinical applications has been limited so far, partly because these require specific expertise and are relatively expensive. Moreover, besides MS other glycoform detection modalities are also suited for clinical implementations, for example, methods based on antibodies or lectins, or aptamers that allow the detection of single molecules (see Fig. 1) (29, 30, 31, 32). For diagnostic purposes, these latter approaches appear attractive for future routine applications, since these assays are relatively easy to perform, interpret and report, and often exhibit a low cost and high throughput. It is noted that such assays may suffer from nonspecific binding and often do not detect one certain antigen (or glycan structure) but rather a heterogeneous mixture of analytes. Accurate quantifications turn more difficult when the analyte that is measured, that is, the measurand, is poorly defined, whereas MS-based strategies carry the advantage of an inherent precise definition of the measurand in multiplexed readouts (33). Recently, it has been demonstrated that MS provides an excellent platform for the development of a calibration system including standardization for apolipoprotein quantifications (34, 35). This potential also holds for glycoform quantifications in clinical applications. So far, MS-based protein glycoanalytical strategies mostly report relative quantifications using carefully designed cohorts as will be exemplified in the next section. Next, the steps will be discussed that lay ahead to further improve quantifications.

## Glycan Markers With Diagnostic Applications

Reliable detection of interindividual differences at the molecular level is a prerequisite for the translation of (glyc)omics markers into precision medicine (36). In a clinical laboratory context, the sensitivity of a diagnostic test is looking at the diseased population and is concerned about the number of false negatives, whereas test specificity addresses the healthy part of the population and is concerned about the number of false positives. In analytical chemistry nomenclature, sensitivity and specificity of a method relate to the limit-of-detection of a certain analyte and its identification precision. For clinical decision-making, the positive- and negative-predictive values (PPV and NPV, respectively) are more relevant parameters. PPV and NPV are the proportions of positive and negative results in a diagnostic test that are true positive and true negative results, respectively. PPV and NPV reflect test performance and depend on the prevalence of the disease that is considered. These are important in case the glyco(proteo)mics test is aimed at early detection in a population screening program. With regard to diagnostic purposes knowledge of protein-specific glycosylation changes in major plasma glycoproteins has been around for a long time. For example, glycosylation changes in alpha-1-acid glycoprotein (AGP, or orosomucoid) have been described for a range of physiological and pathological conditions using rocket electrophoresis, and these decades-old observations just recently experienced a revival with the advent of state-of-the-art bottom-up clinical glycoproteomic studies (37, 38, 39, 40). For example, upon evaluation of derived glycosylation traits in patients with a malignant melanoma, it was found that fucosylated glycans on AGP were upregulated, whereas their nonfucosylated counterparts were downregulated (38). Upon consideration of the total N-glycan profile of AGP, it was reported that N-glycan branching was increased, and sialylation levels were decreased in individuals who are at risk of type 2 diabetes compared to controls (39).

Another early example of a glycoprotein with value for clinical diagnostics is transferrin, be it for the assessment of alcohol abuse through carbohydrate-deficient transferrin (41) or for the diagnosis of CDGs (10). Commonly, the diagnosis of a CDG is performed through the characterization of a sialic acid deficiency by isoelectric focusing of serum transferrin; however, MS-based approaches on released N-glycans or on intact transferrin rapidly seem to replace the gel-based method because these provide information on the specific defect (12, 42, 43). In addition, plasma protein N-glycan profiling has been evaluated in order to obtain more insight on glycosylation changes associated with CDGs (44, 45). For example, in cases of defects in proteins involved in Golgi trafficking (COG5-CDG) or CMP-sialic acid transport (SLC35A1-CDG), lower levels of sialylated structures on plasma proteins were found in patients when compared to healthy controls, with a more pronounced effect for α2,3-sialylation than for α2,6-sialylation (45).

**Fig. 2:**
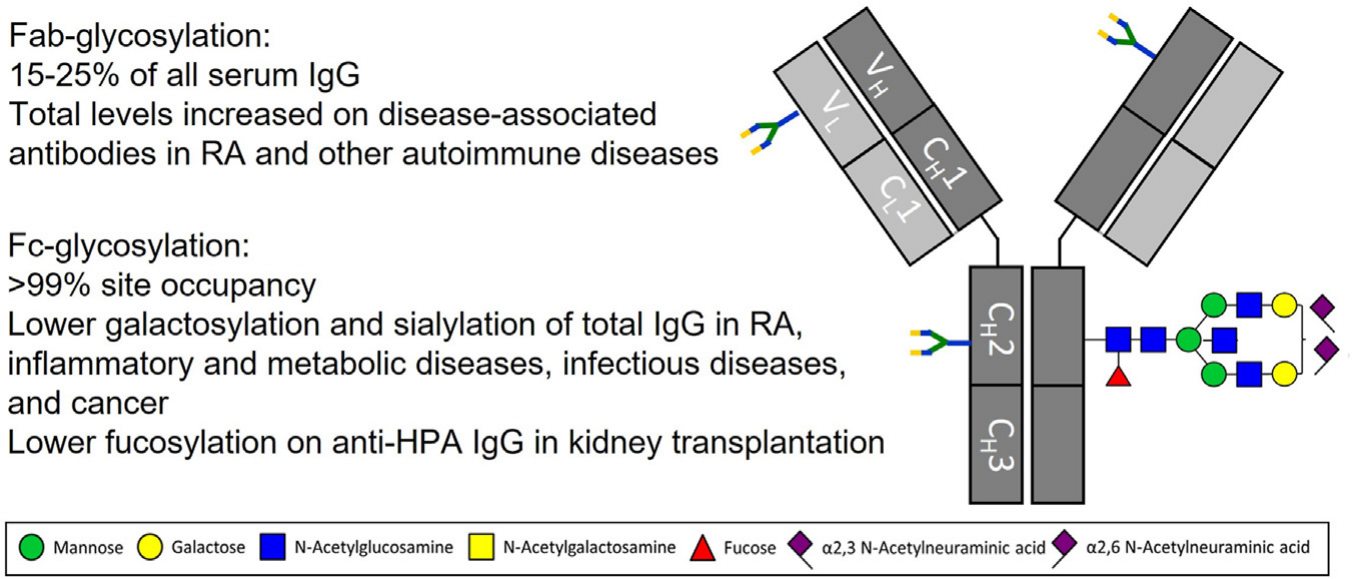
**Summary of IgG-specific N-glycosylation signatures that are associated with various diseases.** Specific glycan structures found on IgG are assigned in (173). IgG, immunoglobulin G.

Immunoglobulin G (IgG) glycosylation signatures have been obtained by various MS methods, all using prior affinity purification, and associations with various diseases are summarized in Figure 2. Low galactosylation has been described for rheumatic disorders (46, 47), as well as low levels of both galactosylation and sialylation, and reported for many inflammatory and metabolic conditions (48). In addition, major variation in IgG glycosylation between individuals occurs with regard to Fc-galactosylation and sialylation at the population level. With regard to Fc-galactosylation levels, it has been demonstrated that these provide a readout for biological age and as such can be used as a “health marker” (49, 50, 51). Also in the case of severe COVID-19, various changes in the IgG glycome composition were observed of which the most prominent one is an increase in agalactosylation of IgG (52). With regard to Fc-sialylation levels, it has been reported that these mediate the anti-inflammatory activity of IgG in intravenous immunoglobulin preparations which are broadly used in the clinics, often as an anti-inflammatory agent in *e.g.*, autoimmune diseases. Furthermore, in terms of the activity of IgG Fc glycoforms, core fucosylation has been found to modulate IgG interaction with CD16 (Fcγ receptor IIIa and IIIb). This finding originates from glycoengineering research of biopharmaceuticals in conjunction with binding studies (53). Subsequently, glycoengineered low-fucose IgG variants have found their way into cancer therapy. Clinical glycoproteomic exploration of the IgG Fc variation regarding fucosylation has only taken off with a delay in relation to its biopharmaceutical exploration and exploitation: A first report of low fucosylation on pathogenic antibodies stems from 2009 (54), and since then various reports have described the occurrence, role, and clinical potential of variations in fucosylation patterns of antigen-specific IgG responses, covering mainly alloimmune diseases and viral infections. Recently, it was found that in patients who received kidney transplantation, afucosylated donor-specific antibodies against human leukocyte antigen (HLA) could serve as a biomarker for antibody-mediated rejection and contribute to pathogenesis (55). Similarly, in patients that received platelet transfusions, composition of anti-HLA Fc-glycosylation profiles could potentially explain the variation in clinical severity (56). With regard to cancer detection and treatment, the possible occurrence and role of low fucosylation on natural or treatment-induced anti-tumor IgG antibodies have hitherto remained unexplored, while one may speculate that low-fucose anti-tumor IgG may contribute to productive anti-cancer immune reactions *via* glycosylation-dependent recruitment of immune cells. Recently, the relationship between human IgG Fc N-glycosylation and a broad range of common diseases associated with changes in the IgG N-glycome was reported (13). In order to conclude whether the relationship between alterations in IgG Fc-glycosylation and a certain phenotype in humans is causal or consequential requires more mechanistic evidence (47).

Recently, Fab glycosylation of anti-citrullinated peptide antigen (ACPA) IgG autoantibodies was introduced as an early hallmark for the development of rheumatoid arthritis (RA) and may have the potential for guiding the pre-disease, possibly targeted intervention to prevent the development of RA (57). Several other autoimmune conditions likewise feature elevated levels of Fab glycosylation on associated IgG autoantibodies, diagnostic as well as therapeutic approaches targeting Fab glycosylation may therefore have broad applicability for a range of autoimmune conditions with pathogenic autoantibodies (58).

In the “hallmarks of cancer” proposed by Hanahan and Weinberg, glycosylation differences were not included yet (59). However, it has become apparent that glycans play a role in each one of these hallmarks. Various studies have shown that glycans drastically affect the function of a glycoprotein and these structures contribute to the malignant phenotype of cancer cells by promoting proliferation, metastasis, and immunosuppression (60, 61, 62). Knowledge on aberrant glycosylation is of key importance in understanding pathological steps of tumor development and progression and consequently became a hallmark of cancer (63, 64). In search for new biomarkers to address the urgent need for new diagnostic tests for the purpose of earlier detection of (recurrence of) various types of cancer, a large variety of exploratory studies has been performed. For example, studies on serum and plasma glycosylation in retrospective cohorts demonstrated that cases with pancreatic cancer (PC) could be distinguished from healthy control individuals (exemplified in Fig. 1 top-right) (65, 66, 67, 68, 69, 70, 71, 72). Using similar glycomics strategies for the purpose of colorectal cancer (CRC) it was found that N-glycome alterations associate with survival and tumor stage (73, 74, 75, 76).

For the purpose of ovarian cancer detection, N-glycome analysis of serum, plasma, ascites, and tissue samples has been performed and overviewed (77). Various studies based on released N-glycans from serum proteins concluded similar trends in patients with ovarian cancer (OC), such as an increase in sialylated structures and core-fucosylated structures, and a decrease in high mannose structures and complex/hybrid structures. More specifically, it was found that in patients with OC, core-fucosylated diantennary mono-sialylated N-glycans were highly abundant, while bisecting diantennary and non-fucosylated N-glycans were highly abundant in healthy patients (78). In another OC study fucosylation and sialylation were found to be consistently higher in serum from patients (79). Upon comparison of benign and healthy samples, it reported that sialylated fucosylated tri- and tetra-antennary N-glycans were highly expressed in early-stage OC, whereas high mannose N-glycans were decreased in patients with early-stage OC (80). Finally, it was found that high mannose, hybrid, and bisecting N-glycans were decreased in OC samples compared to healthy controls (81).

Detection of hepatocellular carcinoma has been pursued through interrogation of the blood glycoproteome initially through total N-glycan release and later *via* a multiple reaction monitoring (MRM) approach (82, 83, 84). This MRM platform monitors 100 glycosylation sites across 50 serum glycoproteins in a commercialized melanoma test and has been used to profile blood glycoprotein from patients with COVID-19 (85). Aiming of improved (*i.e.*, more specific) detection of prostate cancer (PCa) the serum glycome was profiled using lectin microarrays (86). In a foreseen next-generation liquid biopsy PCa diagnostic test, the detection is based on the analysis of the LacdiNAc epitope present on free prostate-specific antigen (fPSA) in a sandwich format using anti-fPSA antibody followed by the application of a specific lectin to glycoprofile only fPSA (87). This example demonstrates the interest to revisit common protein biomarkers that are routinely measured in clinical laboratories in case these consist of proteoforms such as glycoproteins (as will be further discussed in the next section) (88, 89). To this end, a detailed structural analysis of the various glyco-proteoforms of PSA has been reported and the exact pinpointing of which glycoforms are relevant is ongoing (90, 91). For similar reasons, the site-specific N-glycosylation of carcinoembryonic antigen has been studied (92). In this exploratory study, site- and sample-specific variations in glycosylation were found, but follow-up research is needed to fully unravel the relevance and potential of each site. Other examples of routinely tested biomarkers that are actually glycoproteins are mucins (MUC1 and MUC16), human epididymis protein 4, human epidermal growth factor receptor, thyroglobulin, many of the coagulation enzymes, and all immunoglobulin (Ig) isotypes (93, 94). It is emphasized that these laboratory tests result in an overall glycoprotein concentration without assessing glycoprotein-specific glycan features (yet). There is a great opportunity for more specific phenotyping through a detailed understanding of the clinically relevant proteoforms as will be discussed in the next section.

Regarding glycosylation signatures of tumors, it has recently been reported that specific (sialyl-)Lewis core 2 O-glycans differentiate CRC from healthy colon epithelium (95). Interestingly, carbohydrate-antigen 19 to 9, already commonly used as a clinical biomarker for monitoring purposes of PC, is a sialylated Lewis A antigen that is present on various proteins (96). These so-called tumor-associated carbohydrate antigens are increasingly recognized as promising targets for immunotherapeutic monoclonal antibodies (97). These carbohydrates differ from those of the surrounding non-cancerous tissues and contribute to the malignant phenotype of the cancer cells by promoting proliferation, metastasis, and immunosuppression. Interestingly, aberrant N-glycosylation is increasingly studied in cancer tissue samples using imaging MS (98, 99, 100, 101). The use of fresh-frozen tissues for glycan imaging is not necessary, since formalin-fixed paraffin-embedded tissues of patients with OC have been used (102). The imaging-MS strategy has been applied for CRC to study tumor microenvironment and identify prognostic markers for early-stage cancers (103, 104). Using similar glycomics strategies for the purpose of CRC, it was found that N-glycome alterations associate with survival and tumor stage. The potential of glycosylation features to act as cancer biomarkers has also been explored for proteoglycans (105, 106). Profiling of various GAGs was used to predict the occurrence of metastatic clear cell renal cell carcinoma (107). A similar analysis of urine and plasma-free glycosaminoglycan profiles as tumor metabolism markers resulted in multi-cancer early detection of 14 cancer types (6). Using a systems biology pan-cancer design, cancer-specific reprogramming of GAG biosynthesis was identified. Moreover, GAG-derived structures have also been proposed as a biomarker in heritable connective tissue disorders (108).

Next to the development of diagnostic biomarkers, glycoscience has contributed to the finding of new drug targets since glycans decorate all human cells (109). Clinical translation of therapies directed at human glycans has been limited so far, whereas progress has been made in targeting microbial glycans (110, 111, 112). Various glycobiology-targeted therapeutics are currently in clinical trials as overviewed by Bertozzi and Smith (109). Moreover, theranostics—which involves pairing a diagnostic tool to a therapy—has been identified as a particularly promising avenue for clinical translation (109). The development of specific anti-glycan antibodies such theranostics “may be a rapid way of translating discoveries to the clinic and validating targets by ensuring that only patients most likely to benefit from a therapy are treated” (109). For example, several therapeutic modalities are being developed that target cancer-specific glycans including monoclonal antibodies against the tumor-associated carbohydrate antigen sialyl-Tn (STn) (113, 114, 115). These novel modalities open the way to the design of antibody–drug conjugates, immunotherapy, and cell therapy.

## Foreseen Multimarker Panels in Precision Medicine

Biomolecular characterization of a disease is a key component of precision medicine. While a genotype generally refers to a certain risk and a phenotype to an end-point observation, the true actors that may explain (subtle) differences between individuals or changes within an individual are the dynamically altering biomolecules such as proteins, lipids, and metabolites. From a clinical decision-making point of view, the measurement of a single biomarker with an unambiguous concentration appears attractive due to the straightforward cut-off values. However, omics has demonstrated that multimarker readouts provide a more accurate description of an individual's health or disease state (116, 117). Current routine assays are performed in clinical laboratories to determine biomolecule concentrations in body fluids for prognosis, diagnosis, and therapy monitoring of certain diseases and are mostly based on biomarkers that have been established decades ago. New markers are pursued aiming for improved sensitivity and specificity levels of these tests or aiming for new assays that support diagnoses at an early and curable stage of diseases for which medical tests are lacking. For example, a plethora of proteomics discovery studies have been applied to retrospective cohorts (body fluids as well as tissues), and resulting diagnostic protein markers that are currently measured by MS have been overviewed (118). Still, various gaps in biomarker translation exist, both technical and nontechnical in nature, and stringent clinical requirements for the implementation of a specific biomarker into a lab-developed test (LDT) cannot be underestimated (119). Development of a diagnostic test should be performed in a concerted effort between analytical and clinical chemists, specialized physicians and clinicians, and laboratory experts. For this purpose, the use of structured frameworks for test evaluation is recommended, such as the frameworks of the European Federation of Laboratory Medicine, the American Association for Clinical Chemistry, or the International Federation of Clinical Chemistry to furthermore guarantee that tests are developed and implemented that are fit for clinical purpose (120). The collection of clinical evidence for one or more biomarkers requires prior assay validation, transfer, and implementation. Next, the integration of glyco(proteo)mics strategies with data from various diagnostic modalities is key not only in precision medicine but also for predictive and preventative purposes (121, 122, 123). With regard to the applied technologies, in omics studies, it is emphasized that the measurements have focused on biomarker identifications rather than quantifications. The analytical tools have turned very efficient in identifying large numbers, but developments to achieve true quantities have been limited. In a multimarker approach, which takes advantage of the inherent multiplexing character of omics strategies, these translation hurdles may be circumvented. In such an approach, relative quantities of the biomarkers are reported where the aforementioned stringent clinical regulations may not apply. Currently, multimarker panels are pursued in various manners and each of these approaches has the potential to improve current diagnostics and pave the way for translation of molecular profiling in biomarker discovery labs into clinical decision-making. One approach to establishing a multimarker panel consists of a comprehensive omics-list, for example, from glycome analysis or proteome analysis (“proteotype”) in which all species are included in biostatistical evaluations (18, 19, 124, 125). In a second approach, various -omics data are combined resulting in an integrated multiomics dataset (99, 126, 127, 128, 129, 130). In a third approach, a multimarker panel is obtained by mapping various proteoforms of one specific (clinically relevant) protein that is routinely measured in a clinical immunoassay. The term proteoform was coined to refer to a “group of proteins” that point to a single gene (131, 132). A proteoform panel is obtained from an intact protein analysis (top-down) with or without the use of immunopurification ahead of the analysis or is inferred from a profile of corresponding proteotypic peptides (bottom-up) (133, 134). Despite the fact that each protein can appear in a wide variety of proteoforms, and the proteome consequently contains millions of different structures, it can be argued that a limited number of proteoforms is sufficient to decipher a disease phenotype, that is clinically relevant and has potential for patient stratification (88, 89, 99). This new insight is important considering the fact that various routine laboratory tests that determine protein levels for diagnostic and monitoring purposes, as well as patient stratification, actually target glycoproteins and do not assess glycoprotein-specific glycan features (94). All glycomics and glycoproteomics examples that have impacted clinical research so far are based on one of these three multimarker approaches. Moreover, the derived glycosylation traits that consist of various glycoforms with shared structural features inherently have a multimarker character. Interestingly, in the earlier mentioned 2012 roadmap, the authors state that *“Many of the oldest and most widely used clinical tests for cancer, such as those for carcinoembryonic antigen (colon cancer), PSA (prostate cancer), and cancer antigen 125 (ovarian cancer), rely on the detection of glycoproteins. All of these glycoproteins have both altered polypeptide expression and altered glycosylation in cancer. While today’s Food and Drug Administration (FDA)–approved assays rely on detecting only a single biomolecule, it is likely that the true diagnostic power of cancer-specific glycoforms will result from multiplexing several glycoprotein biomarkers.”* Ten years later, with many clinical -omics studies performed, the biomarker community indeed has turned from searching for a single biomolecule (“low-hanging fruit”) to appreciating complex biomolecular panels, and this also holds for glycoproteins. As discussed, the multiplexing character of a glycoproteomics panel may originate from a comprehensive analysis of all glycans (on the proteome) resulting in a so-called glycotype or from an integrated multiomics dataset (or cross-omics) (135), or from an in-depth analysis of all glyco-proteoforms of a single glycoprotein. Each of these approaches requires advanced bioinformatics to enable various stakeholders to review and interpret complex multi-analyte data (136). The development of bioinformatic tools will empower translational researchers to identify applications with clinical utility, followed by communication of complex glyco(proteo)mics data to clinicians in an accessible way that still allows thorough review (137, 138). In parallel to the bioinformatic developments, the transition from a single marker readout to a multimarker panel for clinical purposes requires careful consideration of how quantification and data integration is performed. In order to allow a transition from a single marker readout to a multimarker panel for clinical purposes a careful consideration of how quantification is performed is crucial (139, 140).

## Proposed Tiers for Quantification of Glyco-Multimarker Panels

The development and successful establishment of reproducible glyco-diagnostics in a clinical setting require precise knowledge of the structure of the analyte(s). In a biomarker discovery setup, samples from clinical cohorts are annotated (categorized) in a comparative analysis, whereas in routine practice the assessment of one individual relies on a single measurement. The latter type of measurement requires an effective calibration strategy that consists of both internal standards and characterized and accurately quantified external calibrators (94, 119, 141, 142). With regard to the identification part, in-depth glycan structure elucidations taking into account all isomers are of great value (24, 143, 144). With regard to the quantification part, be it single or multi, the analytical quality should be in agreement with the intended clinical use. This implies that the foreseen assay meets predefined minimal performance goals for bias and imprecision based on the biological variation(s) of the analyte(s) within and between individuals (119, 145). These and other (legal) requirements are clarified in documents from the FDA or the European Medicines Agency for the implementation of an LDT or a new medical test; however, a discussion falls outside the scope of this perspective. Nevertheless, it is important to mention that multimarker development and authorization would become extremely tedious (and slow) when all components of the panel were to be considered a single marker (146). Therefore, to demonstrate scientific validity when aiming for a clinical laboratory test, an alternative development and interpretation process for multimarkers is preferred, for example, with regard to quantification. In precision medicine, it is foreseen that an individual will be followed over various years, thereby providing a “personal baseline” that may not need such stringent criteria as for current single marker tests (116, 147, 148). The state-of-the-art analytical glyco(proteo)mics toolboxes allow for long-term analysis that is needed to perform prospective longitudinal studies. For this purpose, a multimarker readout is powerful due to its inherent relative ratio character. Earlier the quantitative MS-based bottom-up proteomics community proposed a three-tier system for the discovery of protein biomarkers and anticipated translation into a medical test (149). The guidance document provides a good starting point for well-defined analytical validations of glycomics and glycoproteomics assays and their clinical applications. With proper knowledge and use of such assays, further evaluations are granted to provide clinical evidence that is needed as a next step toward adoption in medical laboratories. To this end, the glyco field is encouraged to carefully look at previous efforts to translate protein markers into the clinic and follow similar strategies (150). In the tier document, the quality of protein quantifications is defined for three different groups. A similar hierarchy can be imagined for the quantification of glycan and glycopeptide markers, as well as intact (glyco)proteins that aim for clinical implementation. A Tier 1 assay is suited for accurate, precise, and clinically actionable information for medical practitioners to allow decision-making in the care pathway of patients. An example of a protein that is quantified in a laboratory-developed test is thyroglobulin (151). In a Tier 1 analogy, the levels of glycoproteins and glycopeptides would be measured in an absolute quantitative manner with or without prior enrichment (see Fig. 1). Depending on the use of the assay data, these tests may need to meet the requirements of the Clinical Laboratory Improvement Amendments of 1988 (CLIA), the US Food and Drug Administration (FDA), or the European Medicines Agency (EMA). Stable isotope-labeled (SIL) internal standards are an essential component of a Tier 1 assay. Isotopically-labeled glycans have been shown to improve the quantification of individual glycoforms in released glycan approaches, although it must be noted that these are not as widely available and tunable as SIL-peptides (yet) (152, 153, 154). This is even more the case for isotopically-labeled glycopeptides and glycoproteins, but the current increasing interest in clinical glycoscience will certainly advance the field of synthetic analogues (143, 155, 156).

Alternatively, the application of a multimarker panel offers the opportunity to define one species as a quantifier and anchor the other analytes to this one in a relative manner. For example, when a specific glyco-proteoform panel is of interest, the (total) protein part could be quantified in an absolute quantitative way according to Tier 1 guidance. In a Tier 2 assay, the changes in expression levels of glycoproteins and glycopeptides are determined in a relatively quantitative manner resulting from perturbations such as drug treatment and disease for nonclinical purposes (144). It is recommended to follow this strategy for the validation of potential biomarkers from a discovery (omics-based) study. Samples may be model systems such as cell lines or non-human animals or patient-derived materials including tissues and biofluids. The proteomics guidance states that “*the requirements for analytical validation of Tier 2 assays mirror those of Tier 1 beginning with the minimum requirement that stable isotope-labeled internal standard peptides for each and every analyte peptide be used”*. However, when applying a Tier 2 assay the goal, it is not necessarily to provide actual concentrations at the glycoprotein level but rather measure precisely and consistently relative changes in the levels of large numbers of targeted analytes across samples “*highly purified glycopeptides of known quantity*” can alternatively be used as external calibrants. With proper use of such glycan standards, various quantitative glycomics studies have been performed at a Tier 2 level as well as glycosylation studies on for example immunoglobulins or glycopeptides from clinically relevant proteins. Chemical labeling of glycans has been applied to allow the normalization of glycan intensities in N-glycome as well as glycoproteomics studies (157, 158, 159, 160, 161). The Tier 3 measurements do not make use of labeled internal standards for each analyte that is targeted. As a result, Tier 3 measurements are best suited for comparative, semi-quantitative measurements of glycoproteins in biological systems. The absence of internal standards for each analyte requires the use of additional chromatographic and mass spectrometric information to establish confidence in the identification and measurement of the analytes being targeted. Examples are MS-based profiles of intact transferrin for CDG diagnosis and studies on apolipoprotein CIII (162). Finally, for the purpose of glycopeptide quantification pre-analysis and (glyco)protein digestion requires careful consideration similar to protein quantification strategies (163, 164). From the early days of MS-based proteomics, it is known that proteolysis is often incomplete which obviously will affect quantitative readouts (165). Recently, these quantitative challenges have been addressed for apolipoprotein(a) by detailed evaluation of the digestion kinetics of the quantifying peptides (35).

## Personal Baselines for Personalized Health Care: Concluding Thoughts

Current clinical interpretations of biomolecular readouts are commonly based on a reference range that is defined as the interval of values that is deemed normal for the specific measurement in healthy persons, that is, 95% of values of a reference population fall into this range. A test that is fit-for-purpose should meet predefined analytical performance goals for bias and imprecision based on the biological variations of the analytes within and between individuals. In the era of precision medicine, patient populations are increasingly stratified thereby providing targeted and more efficient treatments and aiming to reduce adverse treatment effects (116, 147, 148, 166). Differences between individuals (biological variation) may be such that they obscure the biomarker signal. To address this personal glycan baselines have been introduced that allow monitoring (subtle), changes within an individual (167, 168, 169, 170, 171). This approach requires multiple time points from individuals in a longitudinal setup that can support clinicians in for example following treatment response and can be of value in earlier detection of disease in the screening of (sub)populations (172). Precision medicine also brings opportunities for the *in-vitro* diagnostics industry by utilizing novel technology platforms and specialized treatments that combine large-scale data with high-end computational analyses. The advances in biomedical and molecular technologies reduced per-individual cost of high-throughput technologies, such as next-generation sequencing and targeted proteomics. These advances bring omics sciences as a feasible approach to unravel molecular patterns of disease and well-being and hence put precision medicine into clinical practice. The availability of an LDT or medical test that allows precise monitoring of treatment response contributes to personalized medicine in general and improved treatment regimens specifically. Although high-end imaging technologies enable accurate clinical staging, these are hardly used to provide information on therapy response and are considered too expensive to be routinely applied in surveillance programs. Molecular markers, such as the here proposed glycosylation markers, have great potential in the development of novel disease stratification tools. In order to implement such tools in the clinic the importance of further improving quantitative performance and transparency of reporting data has been underlined. The foreseen advances will enable the collection of clinical evidence with regard to test effectiveness as required for each LDT. Using these new tools in combination with longitudinal sampling will provide personal baselines for diagnostic and disease monitoring purposes in the near future.

## Conflict of interest

The authors declare no competing interests.
